# Individuals of the common Namib Day Gecko vary in how adaptive simplification alters sprint biomechanics

**DOI:** 10.1038/s41598-017-15459-6

**Published:** 2017-11-15

**Authors:** Clint E. Collins, Timothy E. Higham

**Affiliations:** 1 University of Idaho Dept. of Biological Sciences Life Sciences South 263 Moscow, 83844 Moscow, ID United States; 2 Department of Evolution, Ecology, and Organismal Biology, University of California, 900 University Ave Riverside, Riverside, CA 92521 United States

## Abstract

Locomotion inextricably links biomechanics to ecology as animals maneuver through mechanically challenging environments. Faster individuals are more likely to escape predators, surviving to produce more offspring. Fast sprint speed evolved several times in lizards, including geckos. However, the underlying mechanisms determining performance await discovery in many clades. Novel morphological structures influence these mechanisms by adding complexity to the government of locomotion. Gecko adhesion coevolves with modified muscles, tendons, and reflexes. We explored how the Namib Day Gecko, *Rhoptropus afer*, sprints on ecologically relevant substrates. Locomotion requires that many moving parts of the animal work together; we found knee and ankle extension are the principal drivers of speed on a level surface while contributions to sprinting uphill are more evenly distributed among motions of the femur, knee, and ankle. Although geckos are thought to propel themselves with specialized, proximally located muscles that retract and rotate the femur, we show with path analysis that locomotion is altered in this secondarily terrestrial gecko. We present evidence of intraspecific variation in the use of adhesive toe pads and suggest that the subdigital adhesive toe pad may increase sprint speed in this species. We argue kinematics coevolve with the secondarily terrestrial lifestyle of this species.

## Introduction

Locomotion is fundamental to the ecology of many animals. Effective locomotion mediates the outcome of critical behaviors including migration, mate finding, and predator-prey interactions. Faster individuals often obtain higher social status, produce more offspring, eat more, and are more successful in escaping predators^1–4^. Moreover, animals must move through mechanically challenging environments. But what determines how fast an animal can move in a given environment? Theoretical maximum sprint speed in terrestrial limbed animals is determined by how fast an animal can move its limbs, how long these limbs are, and how much force is applied to the ground during each stride^5^. Yet, beyond the simplicity of moving their legs as fast and as powerfully as possible, the details of how animals move fast enough in natural settings is little-known for many species. Biomechanics is critical to understanding how animals physically propel themselves through challenging environments and is thus critical to understanding ecology. Slight variation in locomotor mechanics can lead to tradeoffs in pivotal ecological interactions, thereby ultimately leading to differential fitness among individuals^6^.

Natural selection acts on individual variation in locomotor performance in a given environment, thereby altering the trajectory of evolution in the suites of underlying traits governing locomotion^7–10^. In some cases, novel morphological structures have evolved, increasing performance of ecologically relevant tasks^11,12^. They profoundly influence diversification by promoting the exploitation of previously unused habitats and by increasing the complexity of functional units often through the addition of morphological components, or by changing the roles and action parameters of a system^8,11,13,14^. Although ‘realizing’ fast sprint speeds results from dynamic combinations of behavioral and morphological components, it is unclear how novel morphological structures influence locomotion and whether individuals vary in their use of these innovations^15,16^. The adhesive system of geckos, which has evolved independently at least eleven times and has been lost nine times, is ideal for addressing such problems as it permits the characterization of how novel structures affect locomotion^17^. Geckos can exploit inclined and inverted surfaces through a functionally integrated locomotor system culminating in morphologically novel adhesive toe pads^18,19^. Subdigital adhesive toe pads are characterized by scansors, bearing arrays of microfibrillar setae (10–100+ μm in length)^20^. Adhesion is achieved by a combination of van der Waals forces and frictional loading^19^.

While adhesion is often considered an evolutionary innovation, the converse to morphological novelty is adaptive simplification, which some gecko clades exhibit^21,22^. Simplification may be equally as rampant and result in consequences as severe as novelty^21^. Morphological novelty is often associated with a selective advantage, yet less is known about the causes and consequences of their secondary reduction or loss^23^. Simplified states of novelties may themselves have advantages in new environments, permitting animals to circumvent the constraints of a morphological novelty^24,25^. Independent reductions and losses of adhesion in a clade of geckos, *Pachydactylus*, leads to elevated rates of morphological and biomechanical evolution, likely due to relaxed morphological constraints, suggesting selection acts against adhesion in some Gekkonid lineages^24,26^.

**Figure 1:**
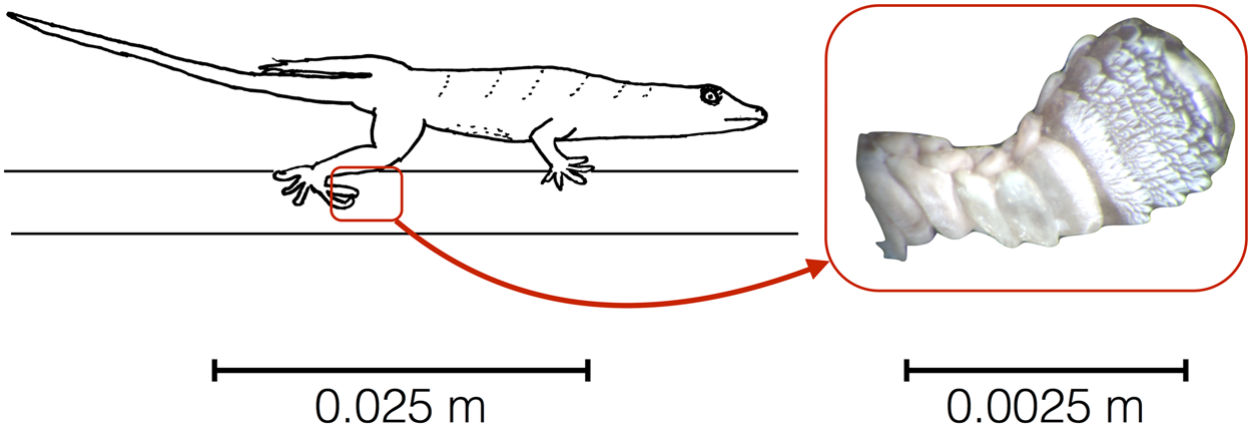
The subdigital adhesive toe pad is lifted from a level surface by hyperextension. Left: Hand drawn image of *Rhoptropus afer* running on a level trackway during mid-stance. Right: The orientation of the subdigital adhesive system in *R*. *afer* during hyperextension.

In some circumstances, adhesion may be detrimental to locomotion. On level surfaces and during the swing phase of each step, the digit tips containing adhesive toe pads are held in hyperextension (Fig. 1). Geckos adhere to substrata by engaging setae via unrolling digit tips downward subsequent to the heel striking the ground^27,28^. Just before lifting the heel, adhesion is released by hyperextending the digit tips. At approximately 10° of incline adhesion is thought to be reflexively triggered. The percentage of strides where adhesion is used then increases to 100% on a 30° incline^29^. Although engaging and disengaging setae is rapid (~20 ms), this process occupies approximately 12.7% of stance time^30^. Therefore, deploying the toe pad imposes a lower limit on the stance phase of locomotion. Hence, this phase of the stride likely restricts an animal’s ability to propel itself forward throughout a stride. In other words, when the toe pad is used, sprint speed may be compromised. This is especially important for secondarily terrestrial geckos that sprint away from predators on terrain that varies in inclines and substrate composition^31^.

Previous accounts of gecko kinematics suggest that geckos detach their toe pads from the substrate before lifting the foot, thereby rendering propulsion through the ankle negligible^27,32^. These authors argue that more proximal musculature would be especially important in geckos because they evolved to climb. Beyond this, a mixture of results from a variety of distantly related lizards suggest that fast cursorial lizards might rely on distal limb function (*e*.*g*. ankle), whereas climbing lizards may rely more on proximal joints and segments (*e*.*g*. femur retraction). Given that adhesion likely evolved in response to a demand for climbing, a key question is whether the relative importance of limb motions changes when geckos become secondarily terrestrial.

Our study focuses on the Namib Day Gecko, *Rhoptropus afer* (Peters 1869). This species sprints up to tens of meters away from simulated predators, and relative to other members of the *Pachydactylus* radiation, accommodates this cursorial lifestyle via a derived morphological pattern, including distally elongated limbs, and reduced toe pad size^26,31,33,34^. We addressed the following questions in this study: (1) how do joint extensions, rotations, and retractions work together to directly and indirectly determine speed, (2) whether and how secondary simplification of the adhesive apparatus affects gecko kinematics, and (3) how do these coordinated contributions work under ecologically relevant conditions?

**Figure 2:**
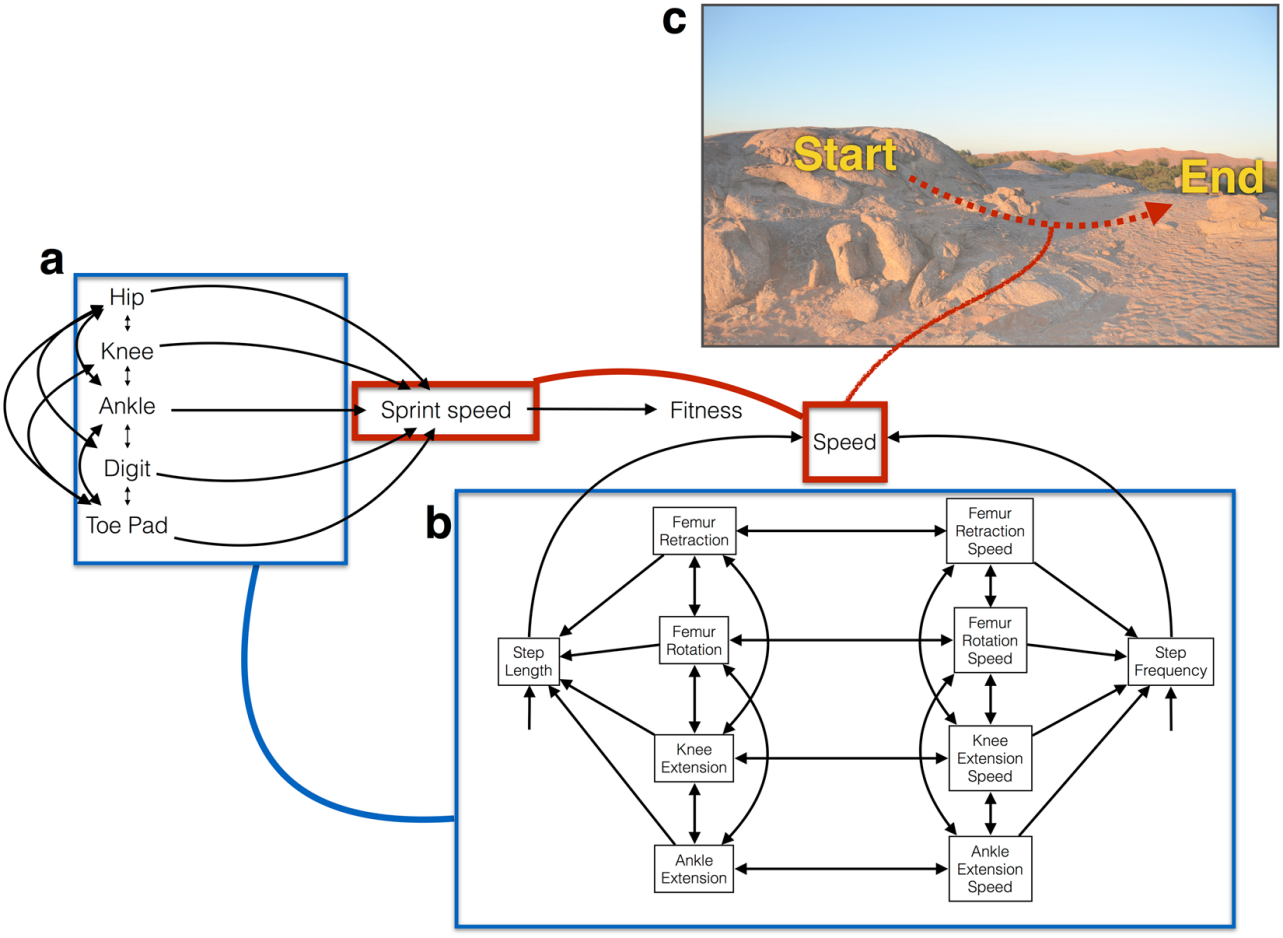
Conceptual model depicting the role of kinematics as the mechanistic linkage between animal morphology and performance in the context of physically challenging habitats. (**a**) We hypothesize that the kinematics studied in this experiment regulate how morphology determines performance in the Morphology → Performance → Fitness paradigm^7^. We focus on one type of performance, sprint speed. (**b**) A path model representing all possible kinematics that could have been measured in this study that lead to step length and step frequency, which in turn lead to speed. We focused on a subset of kinematics that theoretically determine performance on a level and an inclined surface. (**c**) Diagram composed of a photograph from the Gobabeb Research and Training Centre in Namibia and drawing depicting a typical escape path as previously described^31^.

We address our goals using path models, initially developed by Sewell Wright, which are graphical depictions of a hypothesis^35^. They are a framework for developing tests of the direct and indirect effects of independent variables on one or multiple dependent variables. Path analysis simultaneously estimates the *a priori* relationships of all variables to evaluate the effect of multiple predictors on one or more dependent variables^36,37^. We first provide an ecological context for quantifying the mechanical underpinnings of gecko locomotion given the physical constraints on sprint performance (Fig. 2). In this framework, we quantify how kinematics mechanistically link morphology to performance, thereby building on the Morphology → Performance → Fitness paradigm^7^. Kinematics alter how morphology determines performance by facultatively adjusting to the physical demands of the environment and to the behavioral context. In other words, morphological traits can be used in different ways by altering kinematics. We test the hypothesis that the secondary simplification of the adhesive apparatus alters this level of organization in *R*. *afer*. Using path analysis, we asked whether distal or proximal joints and segments determine sprint speed. We tested the hypothesis that distal, not proximal joints, and segments determine sprint speed. Finally, we capitalized on variation in the use the adhesive system to determine if it contributes to locomotor speed, but also if locomotor kinematics, and the differential combination of proximal and distal joints and segments, differ when the adhesive toe pad is in contact with the ground. Throughout the rest of this manuscript, we refer to “toe pad users” and “variable toe pad users”. We qualify this statement because we do not provide direct evidence that adhesion, in the strictest sense, was employed while the adhesive toe pad was in contact with the ground.

## Results

### Principal components, and model fit

Spearman’s (ρ) rank correlation coefficient and Pearson’s correlation coefficient were used to initially describe general relationships between all measured kinematics, as well as the relationship between all measured kinematics and speed (Supplementary Data 1). We computed principal components (PCA) in JMP**®** (Version 10 for Mac. SAS Institute Inc., Cary, NC, USA, 1989–2007) to distill each joint segment and angle motion and the speed of each motion to a representative component. The first principal component summarized 70–89% of the variation in each joint and limb segment. Principal components ameliorate the problem of variance inflation due to multicolinearity^38^ and increase statistical power. Components representing each joint extension and femur retraction and rotation were used in further analysis. Complete Correlation and Variance-Covariance Matrices are available in supplementary information (Supplementary Data 2).

**Figure 3:**
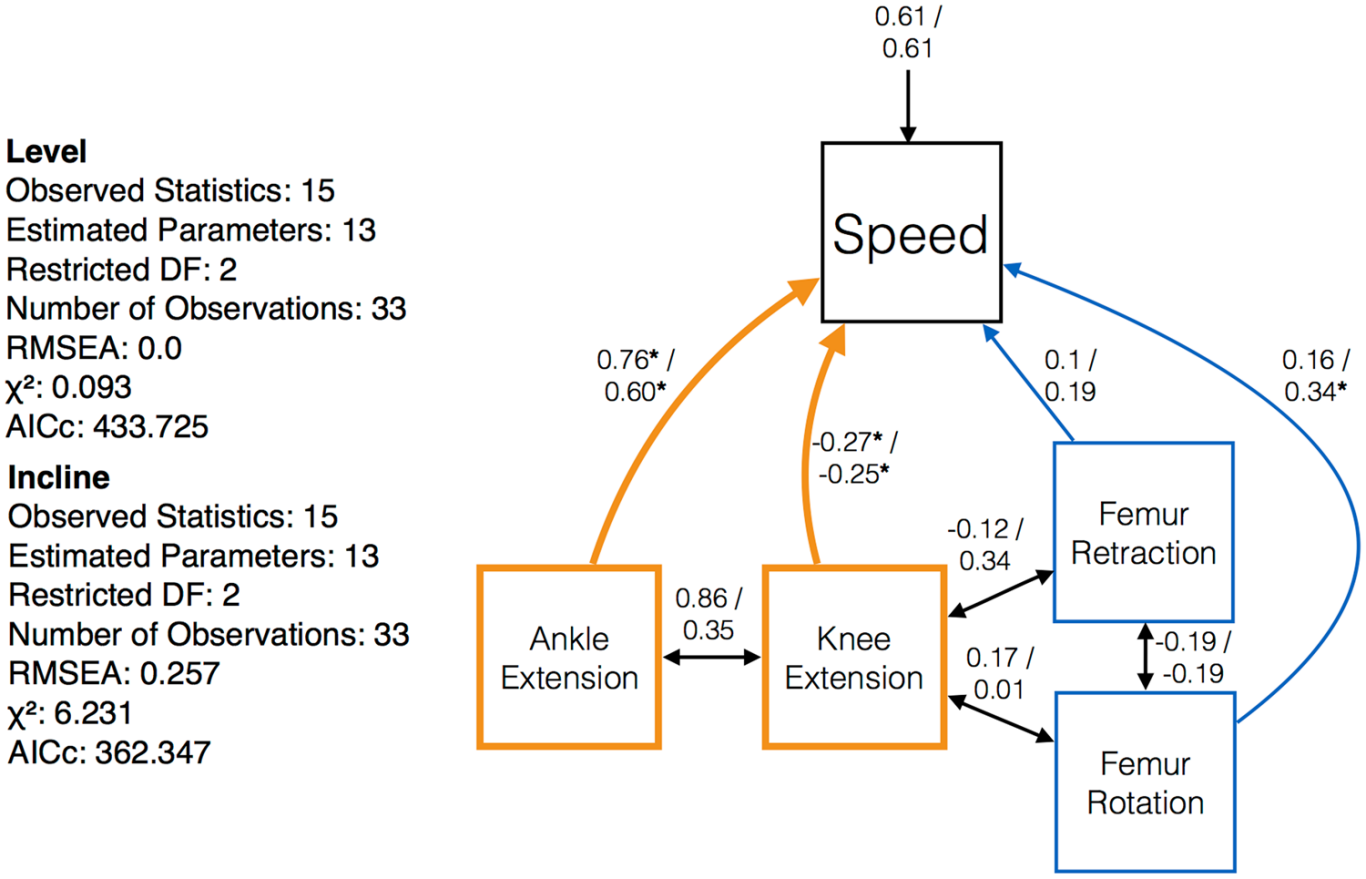
This path model examines how locomotion is governed on response a level and an inclined surface. Numbers on top of the divider represent the relative contribution on a level surface. Numbers on the bottom of the divider represent the relative contribution on an inclined surface, numbers represent relative relationships among traits. Single-headed arrows imply that one variable is causal relative to another. Double-headed arrows (black) indicate a predicted relationship, but that the nature of that relationship is unknown. Asterisks indicate a significant predictor of sprint speed according a Log Likelihood ratio test (p < 0.05).

For the level (0°) treatment, AICc was 452.725, x^2^ was 0.093, the Log Likelihood score equaled −203.862, and RMSEA equaled 0.01 (Table 1, Fig. 3). For the inclined (30°) treatment, AICc equaled 362.47, x^2^ was 6.231, the Log Likelihood score equaled −158.674, and RMSEA equaled 0.257 (Table 1, Fig. 3). Taken together, these model fit indices express a fit decrease in the inclined treatment relative to that of the level surface.

**Table 1:** Sprint speed and the hypothesized kinematic traits exerting control over forward speed in this study, their descriptions, expectations, and outcomes.

**Variable**	**Description**	**Expectation**	**Outcome**	**Statistic**
Sprint Speed	Speed of the center of mass in meters per second.	Sprint speed will decrease from the level to the incline.	Sprint speed decreased from 1.42 ms^−1^ to 1.19 ms^−1^.	p < 0.001
Femur Retraction and Femur Retraction Speed	The 3-D angle (degrees) travelled between the knee and the center of mass during one step and speed of 3-D angle (degrees/seconds) travelled between the knee and the center of mass during one step	Strongest integration with femur rotation. Most important driver of sprint speed. Increase in importance on incline.	Contribution to sprint speed strong, but change in contribution to sprint speed small. Integration with femur rotation greater on incline.	Level RMSEA: 0.265 Level AICc: −183.384 Level −2LLR: 34.43 p < 0.0001 Up RMSEA: 0.212 Up AICc: −253.536 Up −2LLR: 24.37 p > 0.001
Femur Rotation and Femur Rotation Speed	The total amount of rotation (degrees) by the femur during one step and speed of the total amount of rotation (degrees/seconds) by the femur during one step	Strong integration with femur retraction. Second most important driver of sprint speed. Increase in importance on incline.	Femur rotation exhibits stronger contribution on an inclined surface relative to a level surface. Integration with femur rotation greater on incline.	Level RMSEA: 0.204, Level AICc: −204.444, Level −2LLR: 13.8, p = 0.05, Up RMSEA: 0.202, Up AICc: −273.182, Up −2LLR: 24.03, p = 0.001
Knee Extension and Knee Extension Speed	The 3-D extension (degrees) of the knee during one step and speed of the 3-D extension (degrees/seconds) of the knee during one step	Strong integration with ankle; links femur movement to ankle. Distal joints and segments exert less control over speed relative to proximal joints and segments, especially on incline.	Knee extension exhibits stronger contribution on an inclined surface relative to a level surface. Integration with ankle greater on incline. Knee extension speed more integrated with femur retraction speed on incline.	Level RMSEA: 0.465 Level AICc: −21.455 Level −2LLR: 154.66 p < 0.00001 Up RMSEA: 0.396 Up AICc: −194.239 Up −2LLR: 119.3062 p < 0.00001
Ankle Extension & Ankle Extension Speed	The 3-D extension (degrees) of the ankle during one step and speed of the 3-D extension (degrees/econds) of the ankle during one step	Strong integration with knee; Distal joints and segments should be indirectly linked with proximal joints and segments. Show weakest relationship with speed, especially on incline.	Ankle extensor muscle group show greatest contribution to sprint speed in both treatments but relative contribution greater on a level surface compared to incline. Integration with knee greater on incline.	Level RMSEA: 0.58 Level AICc: −85.801 Level −2LLR: 153.73 p < 0.00001 Up RMSEA: 0.417 Up AICc: −118.619 Up −2LLR: 106.926 p < 0.00001

In this table, the amount and speed of movement are grouped in rows for ease of explanation and interpretation. Low p-values (<0.05), derived from a Log-Likelihood Ratio Test, reject a more constrained model in favor of the more complete model that includes the joint movement in question. For example, constraining the ankle joint significantly degrades the model fit on level surfaces and incline surfaces. P-values should be considered along with model fit indices, including RMSEA, which increases as model fit degrades, and AICc.

### What determines sprint speed?

Distal joints and segments, especially the ankle and to a lesser magnitude the knee, are the most important factors in determining forward speed on a level substrate (Table 1, Fig. 3). When running uphill, proximal joints and segments increase in their relative contributions to speed, thereby more evenly distributing how locomotion is achieved. Despite this increase, distal joints and segments still exert the greatest influence (Table 1, Fig. 3).

The effects of all joint and segment movements on locomotion in both treatments are summarized in Table 1. For example, when statistically controlling for femur rotation in the level treatment, model fit degraded: AICc decreased to 444.485, x^2^ increased to 6.854, Log Likelihood decreased to −207.243, and RMSEA increased from 0.01 to 0.108 (Table 1). Although statistically controlling for femur rotation in the level treatment was non- significant (p = 0.08, Table 1), decreases in goodness of fit indices imply biological significance. Statistically controlling for femur rotation in the incline treatment was significant according to a Log Likelihood ratio test (p = 0.03, Table 1), a decrease in AICc to 354.104, an increase in, x^2^ 12.987, and a decrease in the Log Likelihood to −162.052 (Table 1).

Statistically controlling for distal joints and segments by setting all ankle and knee paths equal to zero yielded a significant decrease in fit on the level (Log Likelihood Ratio test < 0.0001) and incline (Log Likelihood Ratio test < 0.0001). Proximal joints and segments significantly affected our model in the incline (Log Likelihood Ratio test = 0.04) but not the level treatment (Log Likelihood Ratio test = 0.1).

### How does toe pad contact affect locomotion?

In our experiment, 15 individuals were obligate toe pad users in the level and incline treatment. Interestingly, 18 individuals were variable toe pad users, where the toe pad was deployed in 19 of the 36 strides on the level treatment. In the incline treatment, 34 out of 46 strides deployed the toe pad on the surface. We focus on the level treatment because the variation in toe pad use was greater. Using this variation, we measured how the use of the digit tip, which bears an adhesive toe pad, affects locomotion.

**Figure 4:**
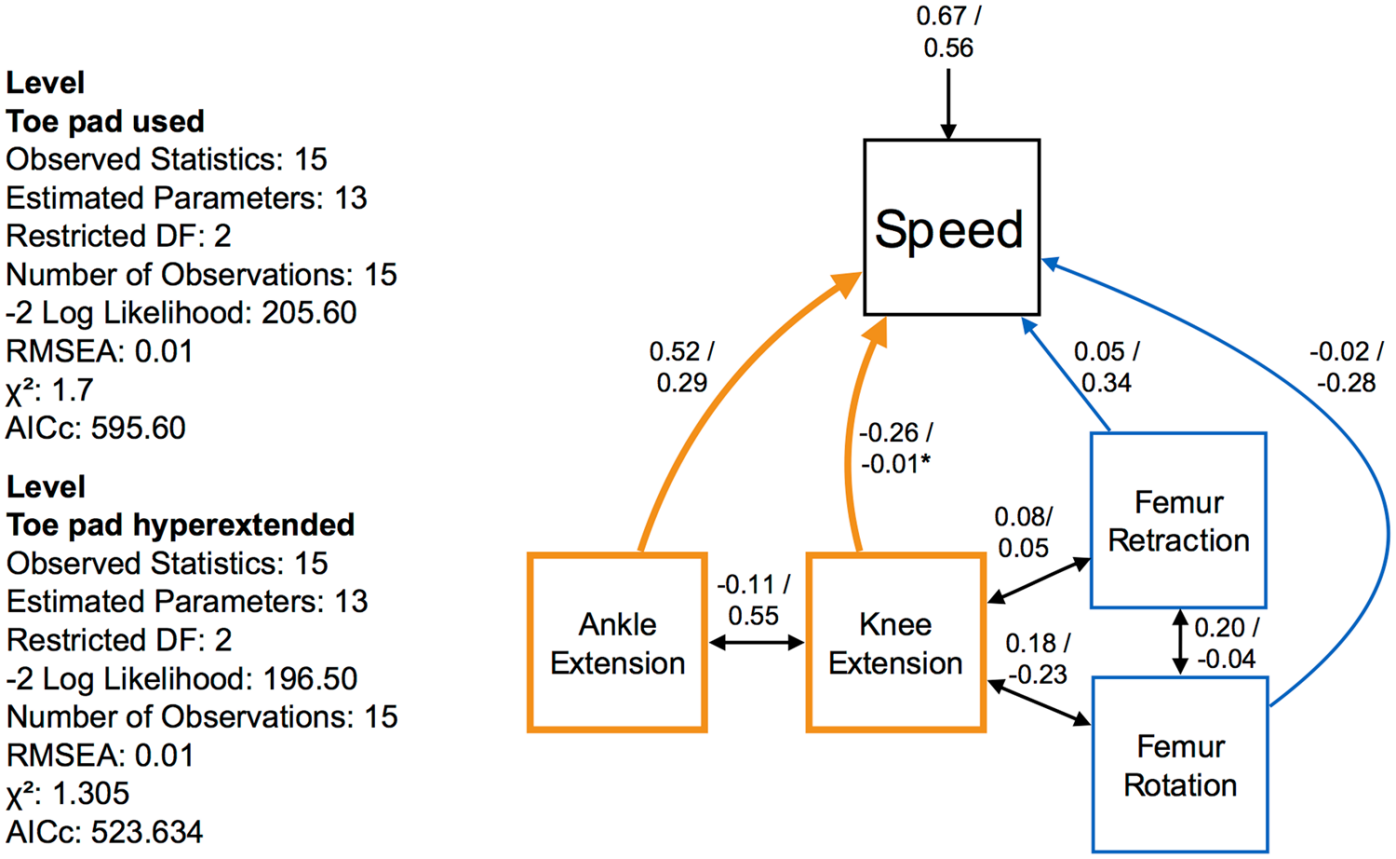
This path model examines how locomotion is governed in response to the use of the adhesive toe pad on a level surface. Numbers on top of the divider represent the relative contribution when the toe pad was deployed, numbers on the bottom of the divider represent the relative contribution when the toe pad was hyperextended.

**Figure 5:**
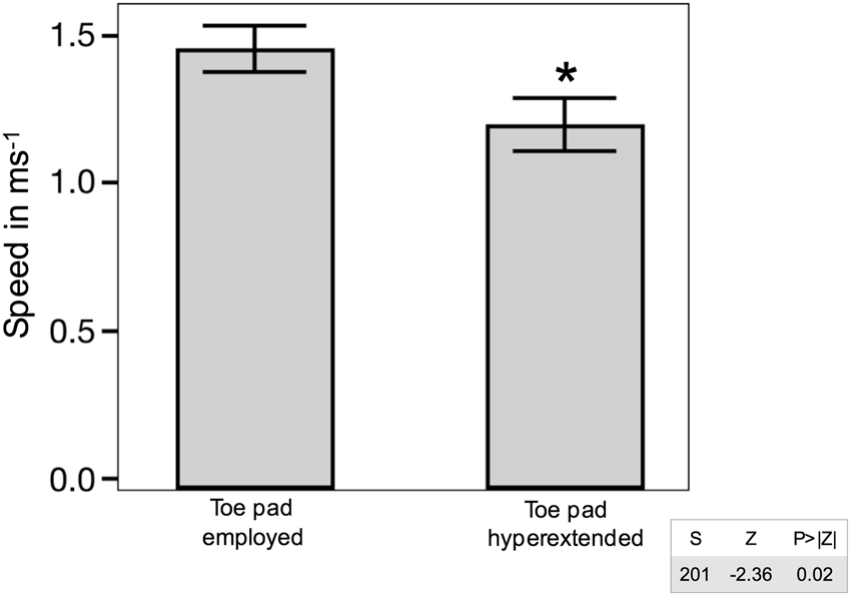
Locomotor speed on a level surface when the adhesive toe pad was deployed on the surface and when the toe pad was hyperextended. Error bars are +/− 1 S.E. *Rhoptropus afer* ran faster when the toe pad was in contact with the surface (Wilcoxon/Kruskal-Wallis).

Ankle and knee extension were the principal drivers of locomotion when variable toe pad users employed the toe pad (Fig. 4). Femur retraction and femur rotation have greater roles when the adhesive toe pads were kept in a hyperextended position in the level treatment (Fig. 4). Locomotor speed in the level treatment was faster in the variable toe pad users when toe pad was unfurled onto the surface (p < 0.05, Fig. 5).

## Discussion

Three important findings are presented in this study. First, morphological adaptive simplification and a cursorial lifestyle correspond with altered kinematics in *R*. *afer* ^23^. This species uses habitat structures quite differently from its climbing congeners, relying on fast sprints to escape over relatively level but variable terrain^10,26,31^. Speed is modulated via ankle extension and knee extension, not femur retraction and femur rotation. *Rhoptropus afer* escapes predators by sprinting over variable but often flat terrain^10,26,31,33^. Altering how speed is governed may release *R*. *afer* from the biomechanical constraints of adhesive apparatus deployment, allowing this species to successfully employ a secondarily terrestrial lifestyle. Second, we found considerable among- and within-individual variation in adhesive toe pad contact. This is a novel finding – previous reports suggest adhesive toe pad deployment does not occur on level surfaces during locomotion and that it is stereotyped in all pad-bearing geckos^29^. We were able to reveal these two findings by using path analysis, an underutilized tool in biomechanics. Finally, we found evidence that *R*. *afer* uses adhesive toe pad deployment to increase its speed on a level surface (Fig. 5). Selection against active hyperextension during the stance phase would increase the length of the out lever, thereby enhancing sprint speed. This is an important finding that suggests a new function for the subdigital adhesive toe pad.

Distal joints and limb segments, especially the ankle, exert the greatest influence over locomotion in the level treatment. This finding is new for geckos. Previous research suggests that geckos propel themselves via specialized climbing musculature located near the center of mass^27,32^. We suggest that adaptive simplification of the adhesive toe pad is associated with the reapportioning of contributions to limb movement in *R*. *afer* ^23^. This is logical given that *R*. *afer* sprints away from predators in a fashion similar to non-Gekkonid cursorial lizards such as *Sceloporus woodi* ^39,40^. Thus, while geckos may have initially evolved musculature and specialized coordination to climb, the diminution of the importance of climbing may be associated with the re-evolution of cursorial style locomotion.

The limbs of terrestrial animals act as a series of levers and fulcra - muscles produce force that is transmitted via tendons to bones (*i*.*e*. the lever), which rotate around a joint (*i*.*e*. the fulcrum). These rotations sum together and are applied to the ground to move the animal^41,42^. How fast or how forceful an animal moves is largely determined by the gear ratio of each joint. The distance from a joint to the application of force, in this case the ground, is known as the out lever. Short out levers amplify the force produced by muscle contraction and are favored where more force is vital for locomotion, such as in digging or climbing. Conversely, long out levers reduce force but amplify velocity and are favored for faster speeds. When geckos lift the most distal portion of their digits from the substrate before full power is reached, they effectively shorten the out lever, thereby reducing their potential for speed^29,30,43,44^. Because some secondarily terrestrial geckos sprint on relatively level surfaces to evade predators, it is logical that natural selection would favor a mechanism to overcome the potential velocity disadvantage associated with a shortened out lever^43^. By reducing the adhesive system, but not losing it completely, *R*. *afer* may be able to capitalize on any available frictional enhancement coupled with the maintenance of the longer out lever. More experiments are needed to reveal the underlying mechanisms of this result. For example, it is currently not known whether the frictional adhesion gained by the contact between the pads and the surface contributes to locomotor speed. In other words, future work should decouple of the roles of lever arms and the potential for adhesion as drivers of sprinting performance.

Path analysis is a powerful method for analyzing the direct and indirect contributions of multiple integrated traits to an outcome^45–47^. Path models are relatively ubiquitous in ecological, evolutionary, and organismal biology^48,49^ yet they are rarely applied in the field of biomechanics. Locomotion, a central theme in biomechanics, is an emergent property controlled and constructed by underlying and integrated suites of traits. Therefore, we used path analysis to assess how locomotion is controlled in *R*. *afer*.

In a meta-analysis, Petraitis *et al*. noted “most published path analyses in ecology should be viewed with extreme caution,” mainly due to problems of variance inflation caused by multicollinearity^38^. Careful planning of path analysis can alleviate these problems^38,50^. Theoretically sound, real models are developed *a priori* with potentially collinear relationships identified by double headed arrows. Therefore, the inherently collinear nature is not only accounted for, but also measured and modeled as part of the theory itself. For example, we used path analysis precisely because the anatomy and mechanics of locomotion are inherently linked and codependent. This codependence was modeled as a part of the hypothesis, rather than as covariates that detract from the realism and interpretation of the analysis. We did not include stride frequency and stride length in our analysis, especially given that an exploratory analysis revealed that the problem of multicollinearity would be exacerbated by including them (Supplementary Data 3). Dropping these variables increased model fit and reduced multicollinearity. While previous researchers argued against dropping variables to ameliorate the problem of multicollinearity, it is theoretically sound to do so here because step length and step frequency are redundant with regard to their underlying predictors^38,50^. The kinematic variables of interest actually create stride length and stride frequency.

Our path model represents the kinematics that propel the animal during the stance phase of locomotion while running straight on a level (0°) and inclined (30°) surface. What remains to be seen is how terrestrial animals navigate uneven and unpredictable terrain, how they maneuver through turns, how they accelerate and decelerate, and how they enter refugia^51,52^. A more holistic picture of locomotion must be quantified to understand the role of biomechanics in evolution and ecology. Using path analysis will help to clarify subtle but crucial biological information implicit in inherently non-independent model systems, including underlying biomechanical constraints responsible for the speed changes under different conditions in our system. Without closely examining kinematics via path analysis, we would not know if speed changes as a result of small changes across multiple joints or because of one large contribution at a single joint. In future, using a path model approach will illuminate which muscle groups are labile, co-dependent, or under evolutionary constraints. This is well understood in fish locomotion and feeding, but under-appreciated in terrestrial locomotion^53,54^.

What might account for the deployment or restraint of the toe pad within each treatment? Because we cannot dissect or decouple adhesion from the kinematic extension of lever arms in this experiment, it is difficult to determine why this is the case. Biologists have long recognized the role of individual variation in intraspecific competition, predator-prey interactions, adaptive syndromes, and speciation^55–58^. *Rhoptropus afer* varies across its range in habitat use during predator evasion, adhesive pad morphology, and the use and coordination of the adhesive toe pad^31^. Given the striking individual and temporal variability in adhesive toe pad kinematics found in this study, more work is needed to construct a comprehensive framework for how natural selection shapes biomechanics. More individuals from each population of this species could be used in a series of experiments to determine whether these differences are related to habitat, genetic, or environment x genetic interactions. Diversifying escape tactics decreases a predator’s ability to predict prey behavior; therefore, individuals should adopt dissimilar escape strategies^59^. It is possible that *R*. *afer* adopts dissimilar toe pad control strategies to vary their use of habitat structures. Whereas some geckos are restricted to specific habitat features, *R*. *afer* has a multitude of substrata available^31,60^.

## Methods

Individuals (n = 33, 3.15–5.2 cm Snout-Vent Length, 2–3.5 grams mass) were collected during November 2012 and June 2013 in Dorob National Park and at the Gobabeb Research and Training Centre. Individual geckos were temporally housed in cloth bags following capture and before experiments began. Geckos were recorded from oblique dorsal and lateral views as they ran down a 1.5 m long × 0.15 m wide raceway composed of 60-grit sandpaper at 0° and 30° inclines. These inclines were chosen as a direct result of the data published in a previous study^31^. The wall of the raceway facing the lateral view camera was clear Plexiglas and the back wall was covered with white copy paper to enhance the contrast of the lizard’s outline relative to the background. All trials were conducted in a laboratory at the Namibia Ministry of Fisheries (http://www.mfmr.gov.na) in Swakopmund, Namibia. We used two high-speed Phantom video cameras to obtain lateral and dorsal views (recording at 500–1000 fps). We calibrated the three-dimensional space using a custom calibration object composed of Lego® blocks placed into the field of view prior to experiments. We conducted three trials per individual per day over a period of seven days. All methods were carried out in accordance with relevant guidelines and regulations and all experimental protocols were approved by the University of California, Riverside Institutional Animal Care and Use Committee under Animal Use Protocol number A20110038E.

Prior to the first running trial, the following were marked with non-toxic correcting fluid and a black fine-tip permanent marker: three equally spaced marks were placed down the midline of the dorsum and marks representing the centers of rotation of the right hip, right knee, and right ankle of each animal. These marks were digitized using DLTdv5 in Matlab 2012b (R2012b The Mathworks Inc, MA, USA)^61^. Following digitization, angles, accelerations, and velocities were calculated for the hip, knee, and ankle joints as well as the center of mass for the entirety of each stride^62^. We only analyzed trials in which an individual ran down the center of the raceway without stopping in the field of view. Data were processed using a custom script written in Matlab (R2012b The Mathworks Inc, MA, USA). Videos were digitized and joint angles and angular velocities for the right hind limb were calculated for 2–5 strides per individual for each treatment. We determined whether or not the toe pad was applied to the substrate (deployed) or held in hyperextension for each step by carefully examining the lateral view of each trial (Fig. 1).

We focused our attention on the stance phase of each stride in order to better understand how propulsion is governed. The following kinematic variables were calculated using joint angle and angular velocities derived from the high-speed video: total ankle extension and ankle extension speed, total knee extension and knee extension speed after the knee flexes during the initial phase of stance, total femur rotation and femur rotation speed, and total femur retraction and femur retraction speed (Table 1). Our initial model included step length and step frequency mediating between underlying kinematics and speed. These two variables were excluded from our final analysis due to their multicolinearity with all kinematics and sprint speed.

### Data analysis

We present separate analyses to determine how functional assemblages contribute to, and determine, forward speed and how toe pad use alters this organization. First, to test the hypothesis that functional assemblages represent a proximal-distal division of labor, values for each individual for each treatment were averaged. Then, averages for individuals for each treatment were log10 transformed in Microsoft Excel**©** to ameliorate any non-normal and skewed data. After transforming data, all kinematics were normally distributed and coefficients of skew were below 1.0. Spearman’s (ρ) rank correlation coefficient and Pearson’s correlation coefficient were generated in JMP**®** (Version 10 for Mac. SAS Institute Inc., Cary, NC, USA, 1989–2007) and used initially to describe general relationships between all measured kinematics as well as the relationship between all measured kinematics and speed (Supplementary Data 1). Principal Components Analysis (PCA) models the variation in a set of variables to reduce the dimensionality of a data set. We used PCA to represent a linear combination of the standardized original variables. We computed PCA in JMP**®** (Version 10 for Mac. SAS Institute Inc., Cary, NC, USA, 1989–2007) to distill each joint segment and angle motion and the speed of each motion to a representative component. The components ameliorate the problem of variance inflation due to multicolinearity and increase statistical power^38^. These components representing each joint extension and femur retraction and rotation were used in subsequent analysis.

We specifically focus on the joint and limb segment motions that power locomotion during the stance phase of a stride. The following variables were used in our path model: Femur Retraction, Femur Rotation, Knee Extension, and Ankle Extension (Table 1). Based on previous studies of lizard locomotion we concentrated on these joints and segments during the stance phase of a stride^63–65^. If the extension of the ankle and knee, both of which are considered distal joints and segments (farther away from the center of mass), exert the most influence on the model, then *R*. *afer* kinematics are derived relative to published data on gecko kinematics. If the rotation and retraction of the femur, a proximal bone, exert the most influence on the model, then *R*. *afer* move forward in a manner similar to other geckos.

We used maximum likelihood to model path coefficients in Ωnyx, a free software program serving as a graphical user interface to facilitate the construction and analysis of structural equation models and path diagrams^66^. The integrated, non-independent nature of kinematics prevents researchers from non-invasively isolating joint and limb segment motions to experimentally determine their relative contribution to locomotion. One merit of using path analysis is the ability to statistically quantify the relative contributions of joints and limb segment motions to forward locomotion via model nesting. Model nesting fixes one or more free parameters, yielding a model that is restricted relative to another^36,37^. Nesting models in this manner statistically ‘fix’ joint and limb segment motions, allowing researchers to interpret their effect on the model under different treatments. Significance of these statistical fixes and their associated pathways are determined by model fit indices (did the model improve, stay the same, or degrade). We focused on Root Mean Square Error Approximation (RMSEA), Akaike Information Criterion (AICc, corrected for finite sample sizes), and log-likelihood ratio tests to inform our analysis. Ideal RMSEA equal 0.01 or less. Our initial models met this criterion. Increases from 0.01 indicated that the model degraded when a joint or limb motion was fixed. AICc increased when model fit decreased, and we used log-likelihood ratio tests to determine if model fit decrease was significant.

We proceeded to test relative contributions to sprint speed by setting each path, one at a time, to zero and then measuring model fit. For ease of interpretation in figures, path coefficients were z-transformed in Ωnyx, setting the variance of all independent variables to one and displaying relative weights among paths. However, all goodness-of-fit and significance testing occurred on non-transformed scores. Single-headed arrows imply that one variable is causal relative to another. Double-headed arrows indicate a predicted relationship, but that the nature of that relationship is unknown.

To assess how the adhesive toe pad works in this system, ninety-three strides were analyzed to determine how speed and kinematics are altered when the toe pad is either held in hyperextension or deployed to the substrate. Twenty-seven out of 47 strides on a level surface involved deployment of the adhesive toe pad. On the incline treatment, 34 out of 46 strides involved deployment of the adhesive toe pad. Of the sample population, 15 individuals always deployed their adhesive system on the level and incline treatment. These individuals are called “obligate toe pad users” throughout this paper. Interestingly, 18 individuals varied in their use of the adhesive toe pad. These are referred to as “variable toe pad users”. Those individuals that varied in their use of toe pads (n = 18) were used in a separate analysis to assess the role of the toe pad in intraindividual locomotor variation and performance. We used used Kruskall-Wallis pair-wise comparisons in JMP® 12.1.0 to test the hypothesis that toe pads enhance sprint speed on the level treatment.

The data that support the findings of this study will be available from 14 March 2018 from the UC Riverside Library and ProQuest. Restrictions apply to the availability of these data, which were used under license for the current study, and so are not currently publicly available. Data are available from the authors upon reasonable request.

## Electronic supplementary material

Dataset 1

Dataset 2

Dataset 3
